# Unidirectional prey–predator facilitation: apparent prey enhance predators' foraging success on cryptic prey

**DOI:** 10.1098/rsbl.2007.0087

**Published:** 2007-04-10

**Authors:** Yixin Zhang, John S Richardson

**Affiliations:** 1 Department of Biology, Texas State UniversitySan Marcos, TX 78666, USA; 2 Department of Forest Sciences, University of British ColumbiaVancouver, British Columbia V6T 1Z4, Canada

**Keywords:** trait-mediated indirect interactions, visual predator, apparent and cryptic prey, cutthroat trout

## Abstract

Food availability can strongly affect predator–prey dynamics. When change in habitat condition reduces the availability of one prey type, predators often search for other prey, perhaps in a different habitat. Interactions between behavioural and morphological traits of different prey may influence foraging success of visual predators through trait-mediated indirect interactions (TMIIs), such as prey activity and body coloration. We tested the hypothesis that foraging success of stream-dwelling cutthroat trout (*Onchorhyncus clarki*) on cryptically coloured, less-active benthic prey (larval mayfly; *Paraleptophebia* sp.) can be enhanced by the presence of distinctly coloured, active prey (larval stonefly shredder; *Despaxia augusta*). Cutthroat trout preyed on benthic insects when drifting invertebrates were unavailable. When stonefly larvae were present, the trout ate most of the stoneflies and also consumed a higher proportion of mayflies than under mayfly only treatment. The putative mechanism is that active stonefly larvae supplied visual cues to the predator that alerted trout to the mayfly larvae. Foraging success of visual predators on cryptic prey can be enhanced by distinctly coloured, active benthic taxa through unidirectional facilitation to the predators, which is a functional change of interspecific interaction caused by a third species. This study suggests that prey–predator facilitation through TMIIs can modify species interactions, affecting community dynamics.

## 1. Introduction

Organisms cope with harsh environmental conditions to exploit available resources for survival. When environmental changes cause food limitation, predators may undergo adaptive foraging-mode switches to alternative prey and even different habitats where food is available (Stephens & Krebs 1987). Since predators' foraging efficiency is determined in part by prey detection (Bond & Kamil 2002), cryptic coloration and low activity of prey decrease the probability of detection by predators (Endler 1978; Merilaita & Lind 2005). However, a dynamic interplay between predators' efficiency and multiple prey species differing in traits of cryptic appearance and activity is not well documented. Trait-mediated indirect interactions (TMIIs) among predator and prey species, which are a functional modification of two-species interactions caused by an additional species' phenotypic traits, can affect food-web dynamics through negative or positive effects (Werner & Peacor 2003). Positive species interactions (facilitation) may have striking influences on community dynamics (Bruno *et al.* 2003; Travis *et al.* 2005).

Prey capture by fish is often initiated by visual detection (Hairston *et al.* 1982) and influenced by behavioural and morphological traits of prey, such as prey crypticity to background (Ruxton *et al.* 2004). Some active benthic insects with visually contrasting coloration are more apparent to predators, which may facilitate the detection of cryptic, less-active prey by providing visual cues to fish. Although some theoretical models have explored predators' probabilities of detecting cryptic prey (Gendron & Staddon 1983; Dukas & Clark 1995), little empirical work has considered interaction between cryptic and apparent prey and its effect on predators' foraging efficiency. Here, we present an experimental study to test the hypothesis that fish foraging success on cryptic, less-active prey can be enhanced by distinctly coloured, active prey.

## 2. Material and methods

East Creek (49°16′ N, 122°34′ W) consists of riffles and pools (width: 0.3–2 m) during dry periods. *Despaxia augusta* (Banks) (Leuctridae) is an actively moving larval shredder stonefly with bright yellow colour defined as ‘distinctly coloured’. *Paraleptophlebia* spp. (Leptophlebiidae) is a relatively inactive larval collector mayfly with mottled grey colour, cryptic to substrate background. Resident coastal cutthroat trout *Oncorhynchus clarki* (Richardson), the only fish species in this stream, is primarily a drift-feeding species. Drift samples were collected (11.00–15.00) prior to experiments using drift nets (mess: 234 μm) at five riffles in a 200 m section (discharge: 0.54 l s^−1^). Five Surber samples (0.095 m^2^) were collected from pools to estimate prey densities. All results were reported as means±1 s.e., and all data for ANOVAs were log_e_-transformed and met ANOVA's assumptions.

### (a) Predation efficiency and prey activity

We conducted field experiments to evaluate the effect of benthic-feeding by cutthroat trout on insect densities when drifting resources were limited in seven parallel, flow-through Plexiglas experimental streams. Each channel (1.5×0.2×0.2 m) had a black bottom and was fed with stream water (0.02 l s^−1^, 10 cm in depth), which was similar to a small shallow pool. Netting (mesh: 234 μm) on each channel's inlet and outlet excluded drift invertebrates and prevented prey emigration. Treatments (each with 10 replicates) were: one trout (fork length, 8–13 cm; weight, 5–20 g; starved 1 day) with 30 mayfly larvae (30M); one trout with 20 mayfly larvae and 10 stonefly larvae (20M+10S); control with only 30 larval mayflies; and control with only 20 mayflies and with 10 stoneflies. Owing to low current velocity (<1 cm s^−1^), no mayflies or stoneflies passed the downstream barrier collected by the outlet netting. Treatments were randomly assigned to the seven stream channels and lasted 20 h (17.00–13.00 PST). Since channel availability was limited, treatments were repeated for 6 consecutive days in late August. We counted prey numbers remaining in the channels after each experiment. There was no significant ‘day’ effect detected for any treatment (ANOVA, *p*>0.05), therefore we did not consider day as a factor in the final analyses.

To determine prey activity level, we conducted behavioural observations in the field (12.00–15.00) in a container with 5 l stream water and with 1 cm^2^ grid on the bottom (45×30 cm). Larvae were held in the stream water for 30 min prior to observation. We observed movement rates of individual mayfly or stonefly both with and without trout present (each *n*=10), because trout odour (chemical cue) may affect prey behaviours (McIntosh & Peckarsky 1996). The trout (10 cm) was held in a corner using a mesh cage. We recorded the distance (grid number) each individual prey moved in 120 s, and noted the cumulative time during which the prey did not move.

### (b) Benthic foraging behaviour of cutthroat trout

In the laboratory, we examined trout foraging behaviour on stonefly and mayfly larvae in the same channel with the stream water (depth: 10 cm; 0.02 l s^−1^) at 10.00–14.00. Trout (10–12 cm) were kept in the stream water (40 l) for 24 h without food (12 : 12 h light : dark cycle). The observation chamber (0.5 m in length) was blocked by 1 mm nets placed at two ends. Trout were observed through a hole on a black curtain that separated the observer and the channel. One trout was placed in the chamber for 30 min for acclimation before the trial. While the trout was held by a 6 mm mesh fence at the downstream side, five mayfly larvae (body length: 6 mm) and five stonefly larvae (7 mm) were simultaneously introduced to the observation chamber. After all prey settled on the bottom, the fence was slowly lifted so that the trout could swim to the section with prey. The foraging behaviour of six individual trout was observed separately, for 15 min each. The first five prey items consumed by each trout were recorded. Prey crypsis and movement patterns in the experimental channels and observation containers did not seem to be affected by the unnatural conditions imposed.

## 3. Results

Drifting invertebrate density in 4 h samples before the experiment was 2.7 individuals/m^−3^ (±1.5, *n*=5). Average drifting biomass of the benthos was 0.039 mg m^−3^ (dry mass ±0.016, *n*=5), which mostly were larval Chironomidae and Dixidae. Only three mayfly larvae (*Paraleptophlebia* spp.) were caught and no stonefly. Mayfly larvae were more abundant in pools (626 individuals/m^−2^±104, *n*=4) than stonefly larvae (87 individuals/m^−2^±21, *n*=4).

### (a) Predation efficiency and prey activity

Mayfly larvae were not only cryptic, but also smaller (body length: 5.6 mm±0.1) than stonefly larvae (8.1 mm±0.2). When drift insects were excluded, cutthroat trout consumed both mayfly and stonefly on the bottom. In controls, prey recovery rate was 95%±1.5 of the 30 prey items. Total prey numbers consumed by fish were significantly different between the two treatments: 30 mayflies (30M) contrasted with 20 mayflies +10 stoneflies (20M+10S) (ANOVA, *F*_1,18_=20.1, *p*<0.001). Within the 30M treatment, only 9.25±1.66 mayflies were consumed by trout, whereas in the 20M+10S treatment, 12±1.31 mayflies and 8.05±0.36 stoneflies (i.e. a total 20.05 prey individuals) were eaten. Since the initial numbers of mayfly larvae differed between 30M and 20M+10S treatments, we scaled the numbers of mayflies to percentages consumed in 30M treatment using a ratio of 20/30. Trout consumption rate on mayflies was 30% higher in the 20M+10S treatment than that in the channels with 30M treatment (*F*_1,18_=12.7, *p*=0.002; figure 1). Trout predation rate on stoneflies was significantly higher than that on mayflies (*F*_1,18_=7.9, *p*=0.012). Linear regression indicated no significant relationship between fish fork length and predation rates on mayflies (*R*^2^=0.22, *p*=0.24) or on stoneflies (*R*^2^=0.01, *p*=0.83) in 20M+10S treatment; for fork length-predation rate (*R*^2^=0.09, *p*=0.44) in 30M treatment.

The movement patterns of prey differed markedly between cryptically coloured mayfly larvae and distinctly coloured stonefly larvae (figure 2). Moving distance of stonefly larvae within 2 min was twice as long as that of mayfly larvae when trout were absent (ANOVA *F*_1,18_=41.7, *p*<0.001), and three times longer for trout presence (*F*_1,18_=29.9, *p*<0.001). Stonefly larvae kept moving during most of the observation periods under both treatments, whereas mayfly larvae moved 27% of the time without trout (*F*_1,18_=33.1, *p*<0.001) and 19% of the time when trout were present (*F*_1,18_=46.5, *p*<0.001).

### (b) Benthic foraging behaviour of cutthroat trout

Four of the six trout ate active stoneflies first. Other two trout captured mayflies first, because the fish easily detected these two mayflies, in which one swam into the water column and another moved close to the fish. After successfully finding and consuming apparent prey items, the fish quickly learned to detect moving prey and searched on the bottom, and successfully located and consumed cryptic, less-active mayflies. In terms of the first five prey items consumed, the trout ate more apparent active stonefly larvae than cryptic, less-active mayfly larvae (ANOVA *F*_1,10_=16.4, *p*<0.01).

## 4. Discussion

As a common attribute of predator–prey interactions, TMIIs have significant trophic effects that change species interaction strength, modify community structure and influence population dynamics (Schmitz *et al.* 2004; Preisser *et al.* 2005). While isolated in pools in dry seasons, drift-feeding cutthroat trout consumed benthic prey, which increased trout energy gain and fitness in the stressful environment (Stephens & Krebs 1987). The benthic predation success of the trout depended on benthos' morphological and behaviour traits. The apparent, active prey increased the vulnerability of the cryptic, less-active prey, by supplying a visual cue to trout and alerting the predator to increase effort to search for benthic prey. Such prey–predator facilitation is one form of trait-mediated indirect effects (TMII). However, the cryptic prey vulnerability change did not improve apparent prey's survival as suggested by theory of apparent competition (Abrams 2004).

Although prey often modify their behaviour to respond to predator presence (Sih & Christensen 2001), behavioural changes were not observed for either cryptic or apparent prey, which could be because the trout-bearing stream water already contained enough fish odour. However, trout showed adaptive behavioural modification to search for cryptic prey, after learning apparent prey's visual cue to improve its limited attention. The limited attention on target detection is a key cognitive constraint for detecting benthic cryptic prey (Dukas 2004). Despite the ecological importance of cryptic–apparent prey interaction, how predation efficiency is affected by cryptic and apparent prey has not been quantified by any empirical investigation. This study demonstrated unidirectional apparent prey–predator facilitation through TMIIs, an unreported type of interspecific interaction. Such unidirectional prey–predator facilitation may have an important influence on species interactions, affecting community dynamics. Future work on optimal foraging theory should consider dynamic interplay effects of cryptic–apparent morphological and behavioural traits on predator foraging.

## Figures and Tables

**Figure 1:**
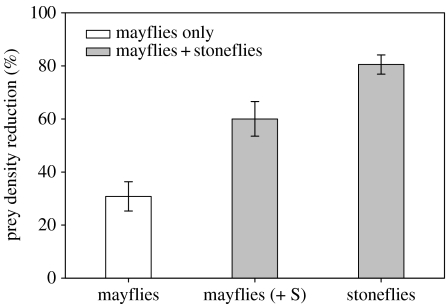
The predation rates (mean %±1 s.e.) of cutthroat trout on mayflies and stoneflies under two treatments: 30 individuals of mayfly larvae (*n*=10) and 20 mayflies (M) +10 stoneflies (S) (*n*=10).

**Figure 2:**
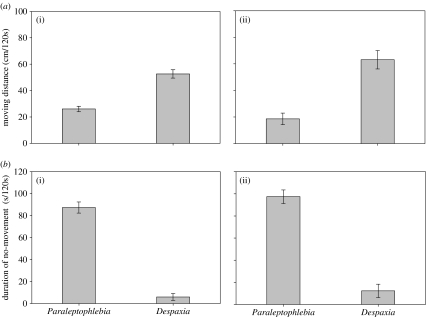
(*a*) Moving distance ((i) no trout and (ii) with trout) and (*b*) duration of no movement ((i) no trout and (ii) with trout) in 2 min by mayfly larvae (*Paraleptophlebia* spp.) (*n*=10) and stonefly larvae (*D. augusta*) (*n*=10). Error bars: ±1 s.e.
